# Supplementary material for: Repulsive vs Attractive Crowding Distinctly Regulate TDP-43 Condensates through Region-specific Structural Dynamics
Source: JACS Au. 2025 Oct 9;5(10):4916–35. doi: 10.1021/jacsau.5c00876 (PMC12569666; doi:10.1021/jacsau.5c00876)
Supplement: Supplementary file 1 [file au5c00876_si_001.pdf]

Supporting Information
for
Repulsive vs. Attractive Crowding Distinctly Regulate TDP-43 Condensates through Region-Specific Structural Dynamics

Guoqing Zhang[1,§], Cibo Feng[1,§] and Xiakun Chu[1,2,*]

[1]Advanced Materials Thrust, Function Hub
The Hong Kong University of Science and Technology (Guangzhou)
Guangzhou, Guangdong 511400, China

[2]Guangzhou Municipal Key Laboratory of Materials Informatics
The Hong Kong University of Science and Technology (Guangzhou)
Guangzhou, Guangdong 511400, China

[*]Corresponding author: xiakunchu@hkust-gz.edu.cn

[§]Guoqing Zhang and Cibo Feng contributed equally to this work.

Tables and additional figures

Table S1: Key simulation parameters.

**Quantity**	**Symbol (units)**	**Value**
Force field / interactions		
Bond spring constant	k_b (kcal mol^{-1} nm^{-2})	1000
Bond equilibrium length	r^0 (nm)	0.382
Interaction energy scale	ε (kcal mol^{-1})	0.2
Relative permittivity	ε_r (-)	80
Crowders		
Crowder mass (PEG1500-like)	(amu)	1500
Crowder bead radius	r_c (nm)	0.8
Crowder volume fraction	$C_{\text{Rep}}, C_{\text{Att}}$ (-)	see text (e.g., up to 0.40)
System setup		
Single-chain box (cubic)	(nm^3)	$25 \times 25 \times 25$
Dimer confinement (spherical)	(nm^3)	$\frac{4}{3}\pi(12.5)^3$
Phase coexistence box (rectangular)	(nm^3)	$15 \times 15 \times 50$
Chains in phase coexistence box	N_{chain} (-)	100
Simulations		
Temperature replicas (single/dimer)	N_{rep} (-)	32
REMD swap attempt interval	(ps)	10
Trajectory per replica (single/dimer)	(μs)	10
Phase coexistence trajectory length	(μs)	5
Langevin damping (single/dimer)	γ (ps)	1
Langevin damping (LLPS)	γ (ps)	1000

Table S2: Symbols used in the main text and Supporting Information.

**Symbol**	**Definition**
C_{Rep}	Volume fraction of repulsive crowder
C_{Att}	Volume fraction of attractive crowder
$\rho(z)$	Mass density profile along z (protein or crowder)
ρ_h	Density of the high-concentration phase (condensate core)
$\rho(r)$	Radial density function
R_g	Radius of gyration
MSD	Mean square displacement for single-chain
D	Diffusion coefficient for single-chain
φ	Angular deviation relative to the z-axis
N_c^{inter}	Average inter-chain residue-residue contact number
N_c^{intra}	Average intra-chain residue-residue contact number
n_c^{inter}	Average inter-region contact number (e.g., helix-IDR, IDR-IDR)
$\mathscr{L}$	Normalized autocorrelation function of n_c^{inter}
τ	Contact relaxation time (from autocorrelation fits)

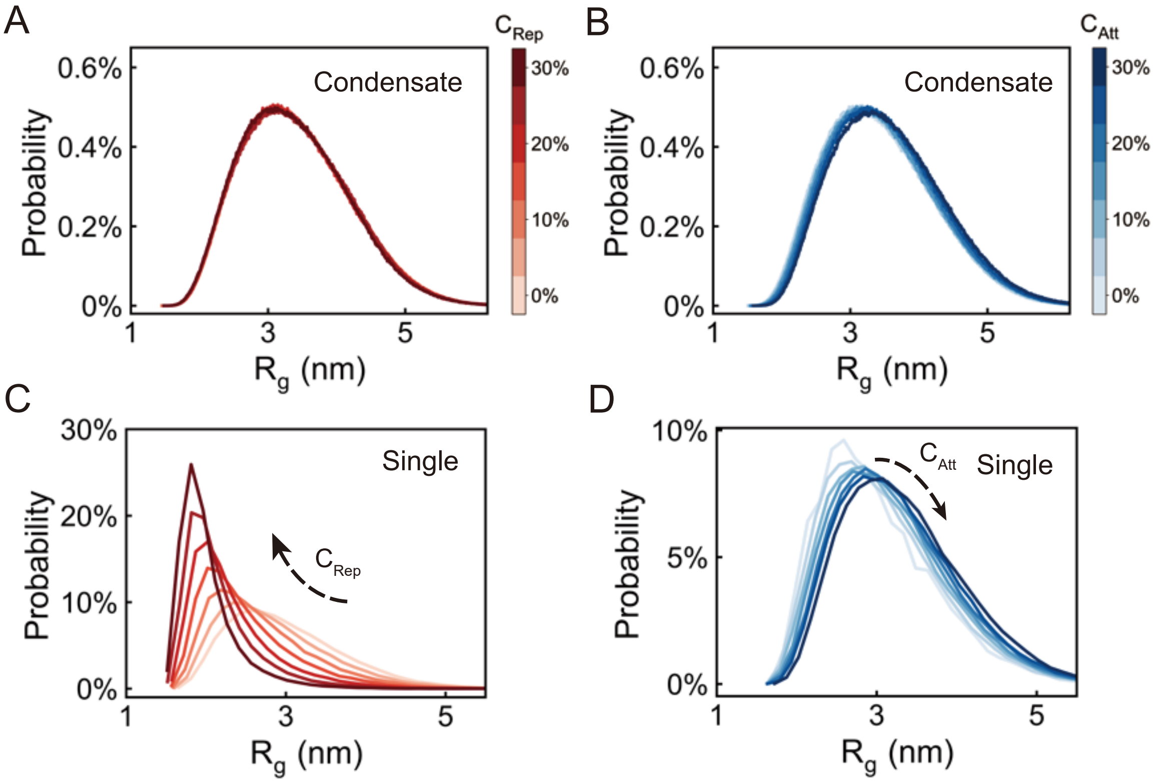

Figure S1: Distributions of the radius of gyration (R_g) of TDP-43 CTD under different crowding conditions. (A, B) R_g distributions for TDP-43 CTD chains within condensates in the presence of (A) repulsive and (B) attractive crowders under varying concentrations. (C, D) R_g distributions for single-chain TDP-43 CTD in the presence of (C) repulsive and (D) attractive crowders under varying concentrations. Each curve represents the probability distribution of R_g sampled from simulations at the specified crowder concentration.

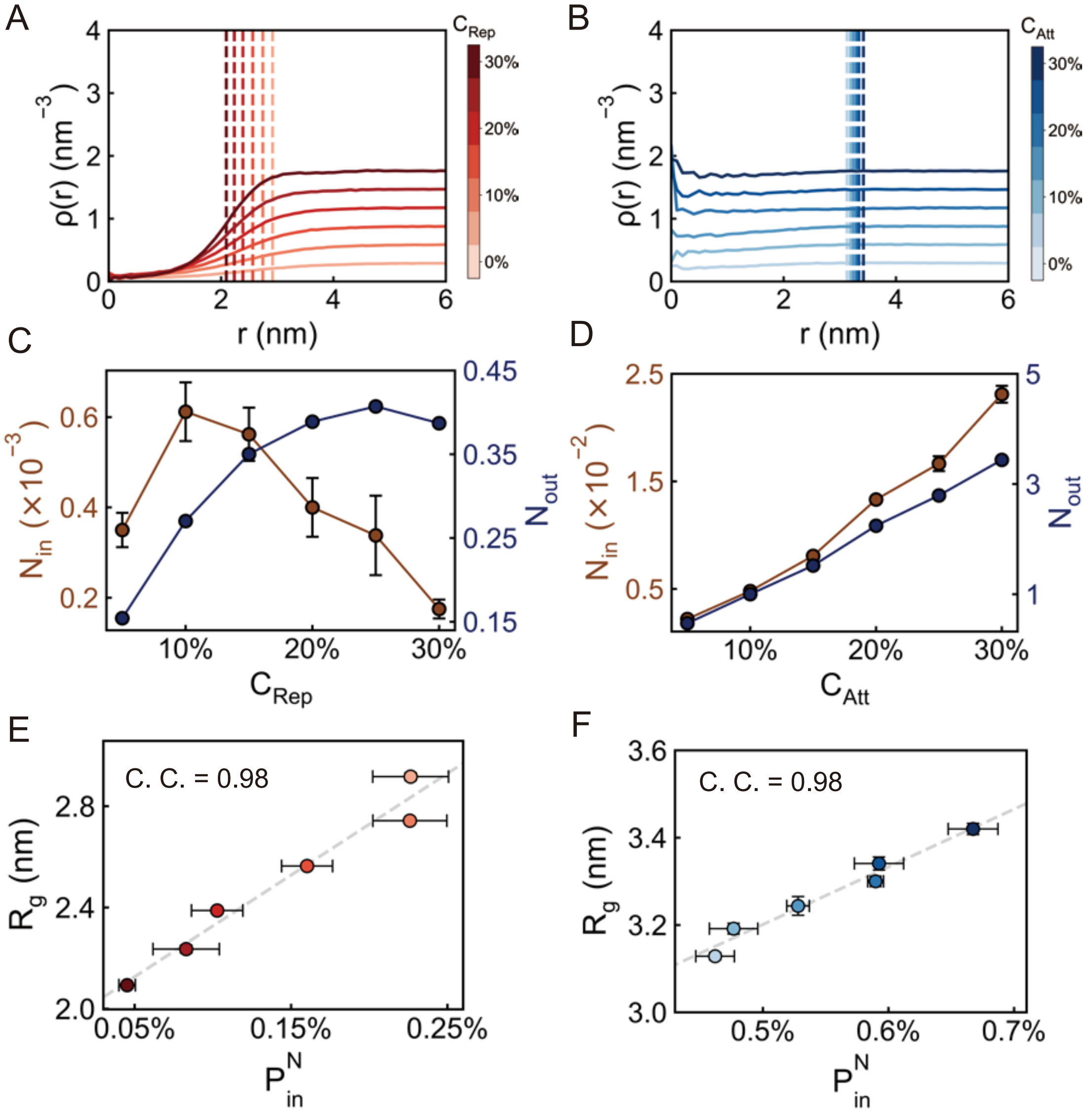

Figure S2: Radial density function (RDF) of crowders relative to the TDP-43 CTD center of mass in the single-chain state under different crowding conditions. (A, B) RDF profiles of crowders in the presence of (A) repulsive and (B) attractive crowders under varying concentrations. Dashed lines denote the average R_g regions of the TDP-43 CTD chains under corresponding conditions. (C, D) Total number of crowders located inside (N_{in}) and outside (N_{out}) the average R_g region of the TDP-43 CTD chain in the presence of (C) repulsive and (D) attractive crowders under varying concentrations. (E, F) Correlation between percentage of crowders located outside (P_{in}) the average R_g region of the TDP-43 CTD chain and the R_g of the single TDP-43 CTD chain in the presence of (E) repulsive and (F) attractive crowders under varying concentrations. P_{out} is calculated as $N_{\text{out}}/(N_{\text{in}}+N_{\text{out}})$ from (C, D). Dashed lines are provided as visual guides to highlight the trends with the correlation coefficients calculated. In panels (C-F), data points represent means $\pm$ standard errors computed from 5 equal partitions of the total dataset.

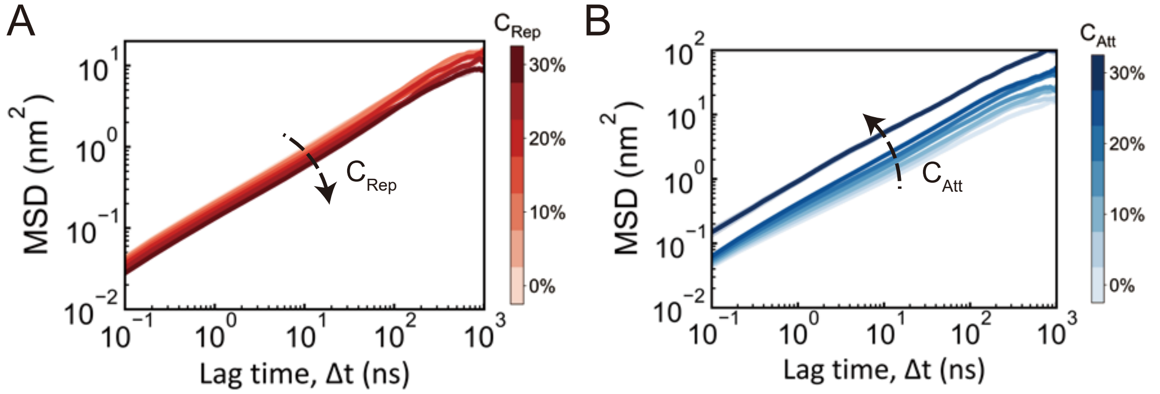

Figure S3: Mean square displacement (MSD) of TDP-43 CTD within condensates under varying concentrations of (A) repulsive and (B) attractive crowders. Data represent means ± standard errors computed from 5 equal partitions of the total dataset.

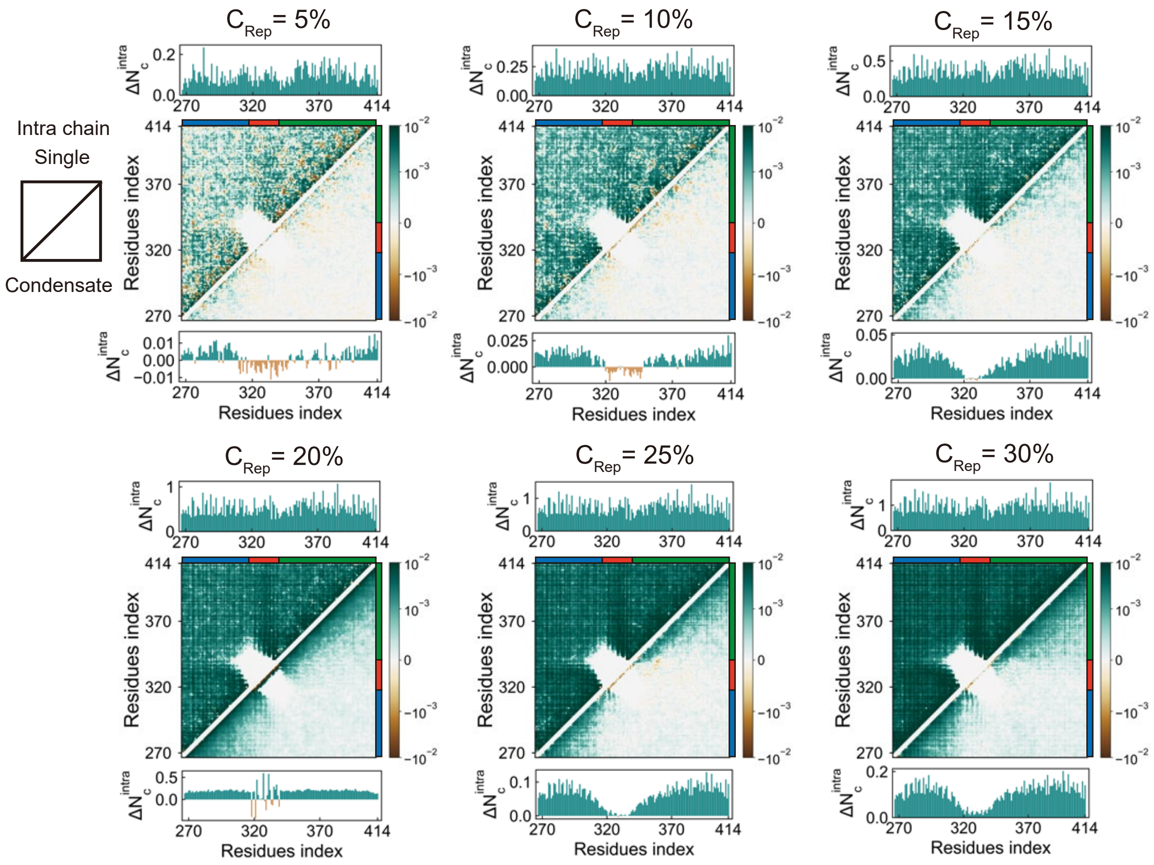

Figure S4: Differential intra-chain residue-residue contact probability maps of TDP-43 CTD under varying concentrations of repulsive crowders. Each map shows changes in contact probability relative to the free condition.

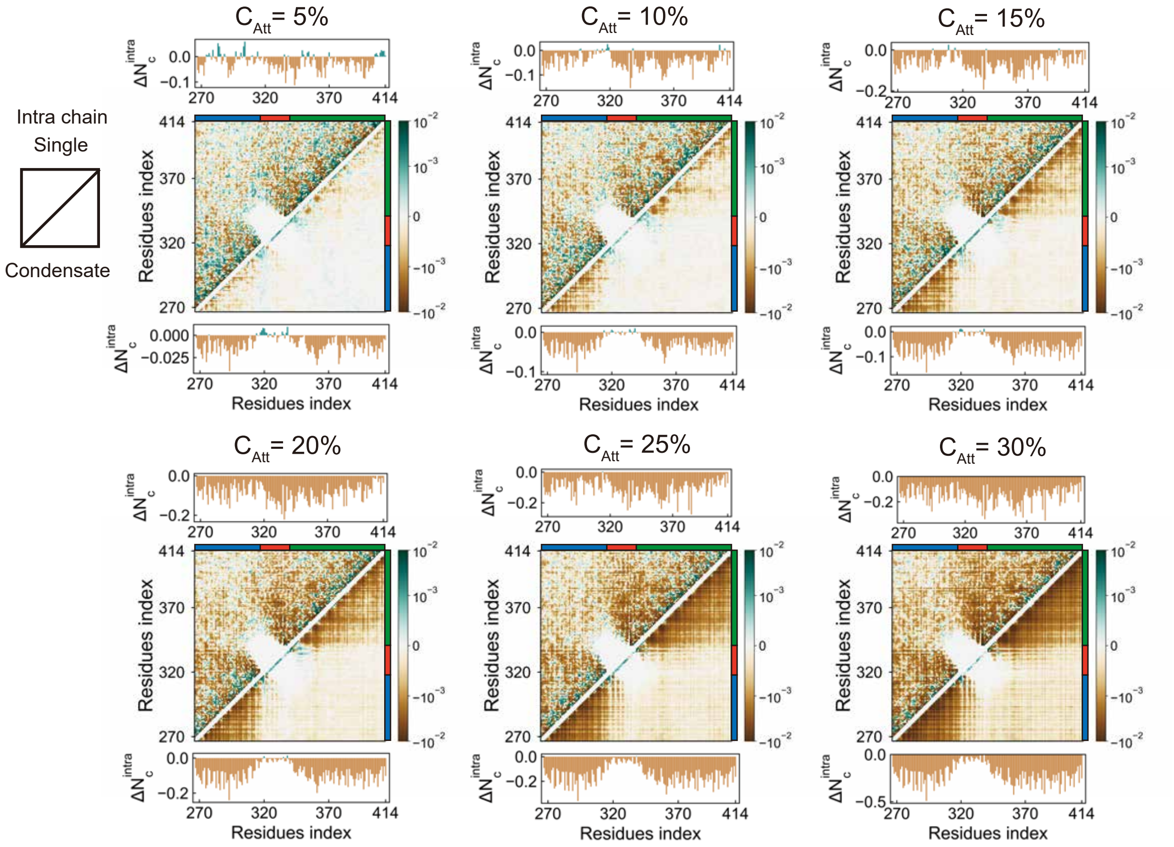

Figure S5: Differential intra-chain residue-residue contact probability maps of TDP-43 CTD under varying concentrations of attractive crowders. Each map shows changes in contact probabilities relative to the crowder-free condition, following the same format as Figure S4.

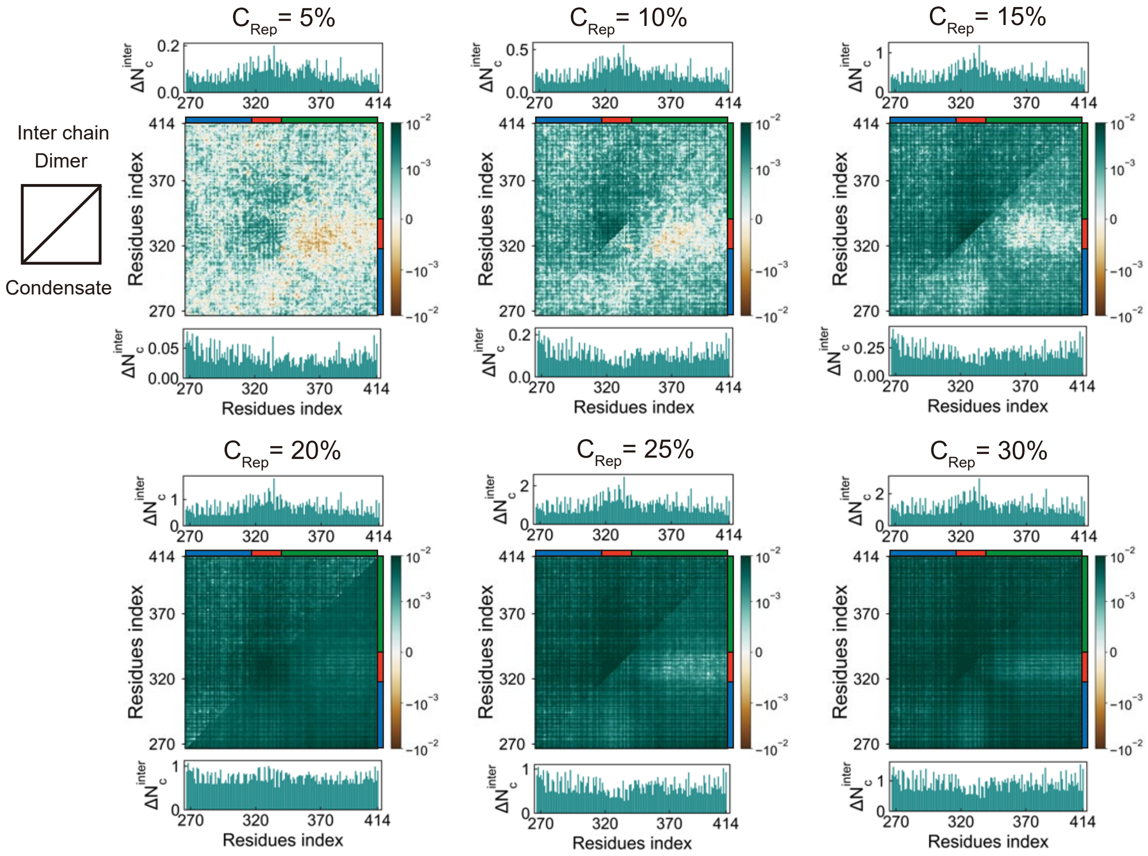

Figure S6: Differential inter-chain residue-residue contact probability maps of TDP-43 CTD under varying concentrations of repulsive crowders. Each map shows changes in contact probabilities relative to the crowder-free condition, following the same format as Figure S4.

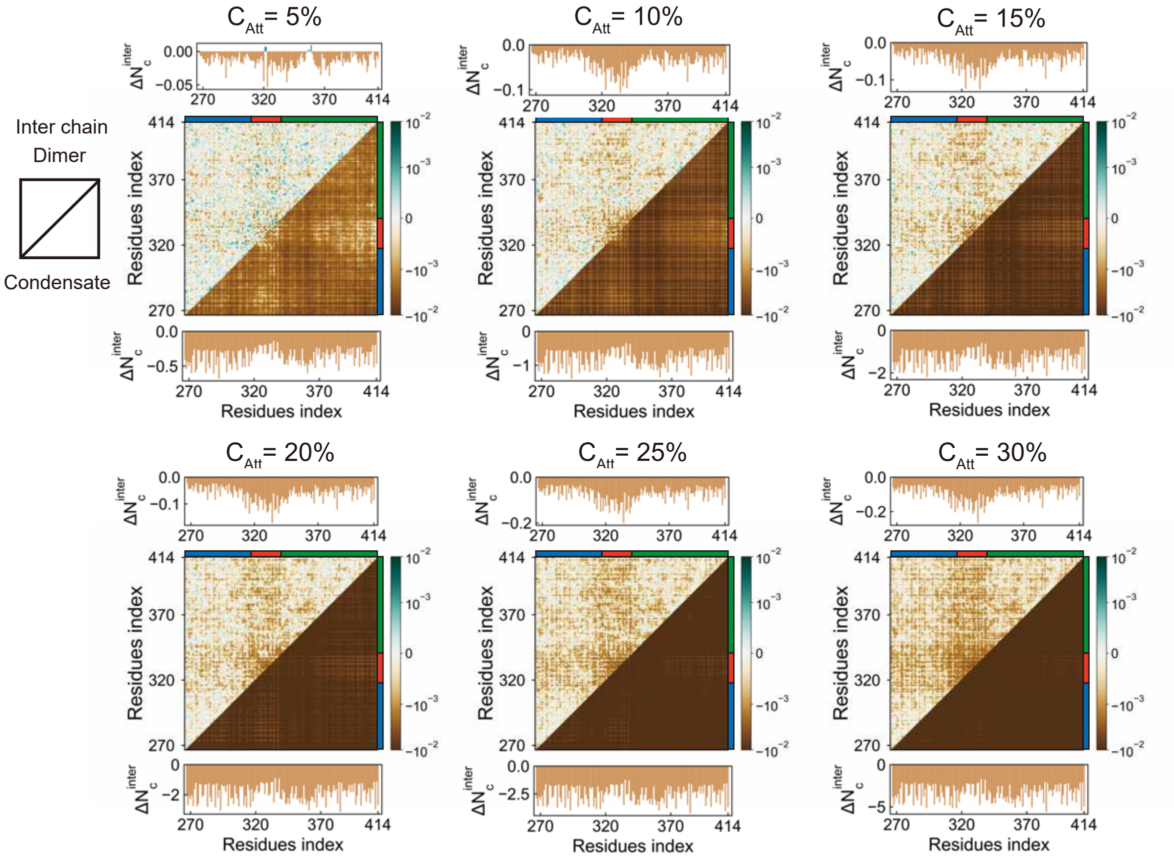

Figure S7: Differential inter-chain residue-residue contact probability maps of TDP-43 CTD under varying concentrations of attractive crowders. Each map shows changes in contact probabilities relative to the crowder-free condition, following the same format as Figure S4.

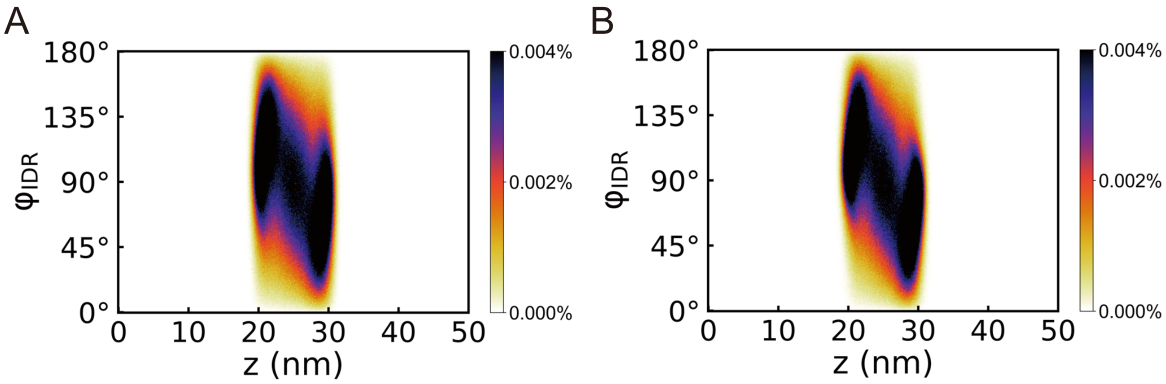

Figure S8: Region-specific orientation angle distributions of TDP-43 CTD IDR segments within condensates under crowder-free conditions. Results were calculated based on (A) the random-selection method and (B) the center-based selection method. In the center-based approach, the representative vector $\vec{r}$ is defined as the vector connecting the geometrical center of the first $(0, L/2]$ and second halves $(L/2, L]$ for IDR2 and as the vector connecting the geometrical center of the second $(L/2, L]$ and the first halves $(0, L/2]$ for IDR1, where L is the length of the corresponding segment.

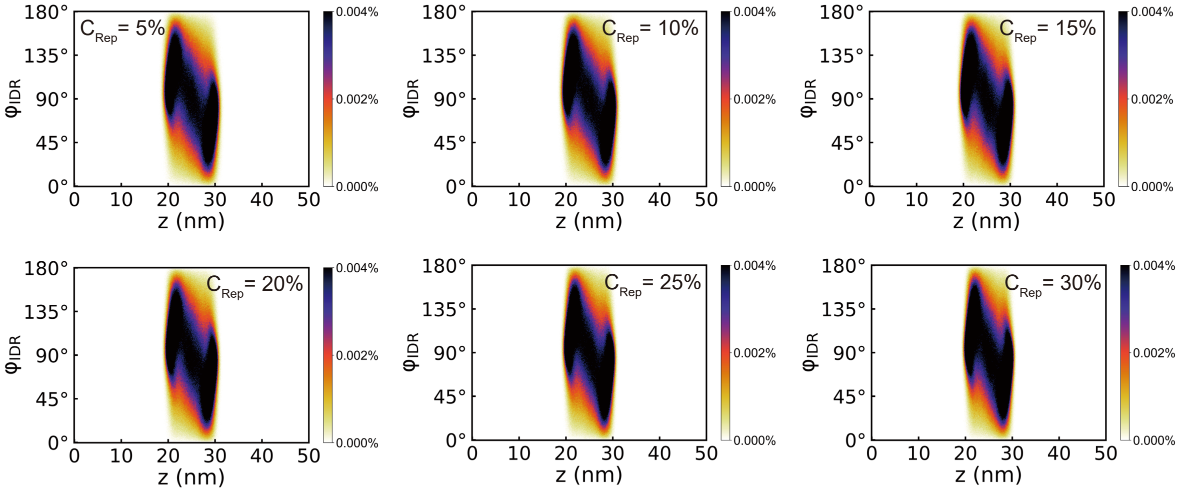

Figure S9: Region-specific orientation angle distributions of TDP-43 CTD IDR segments within condensates under varying concentrations of repulsive crowders. Results were calculated based on the center-based selection method.

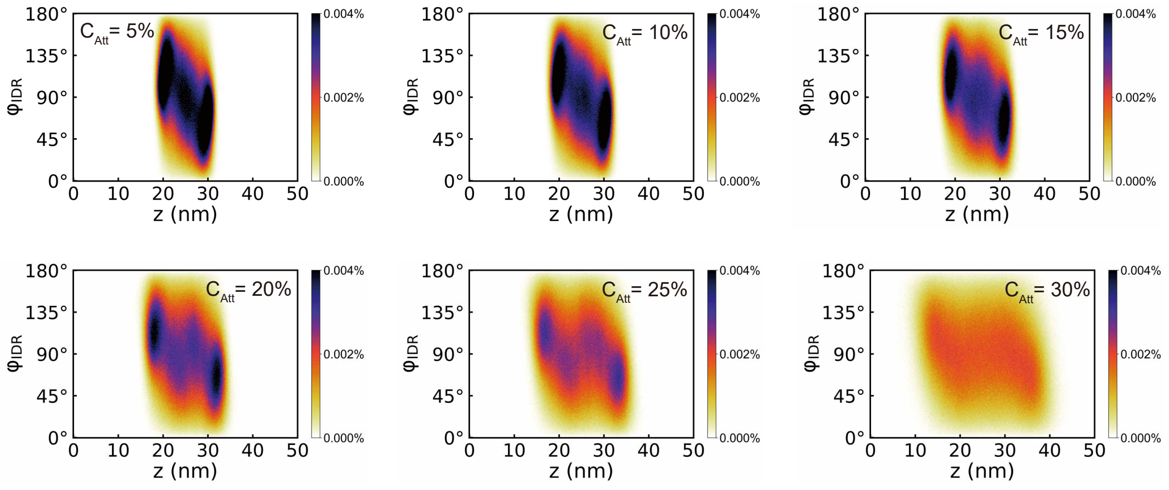

Figure S10: Region-specific orientation angle distributions of TDP-43 CTD IDR segments within condensates under varying concentrations of attractive crowders. Results were calculated based on the center-based selection method.

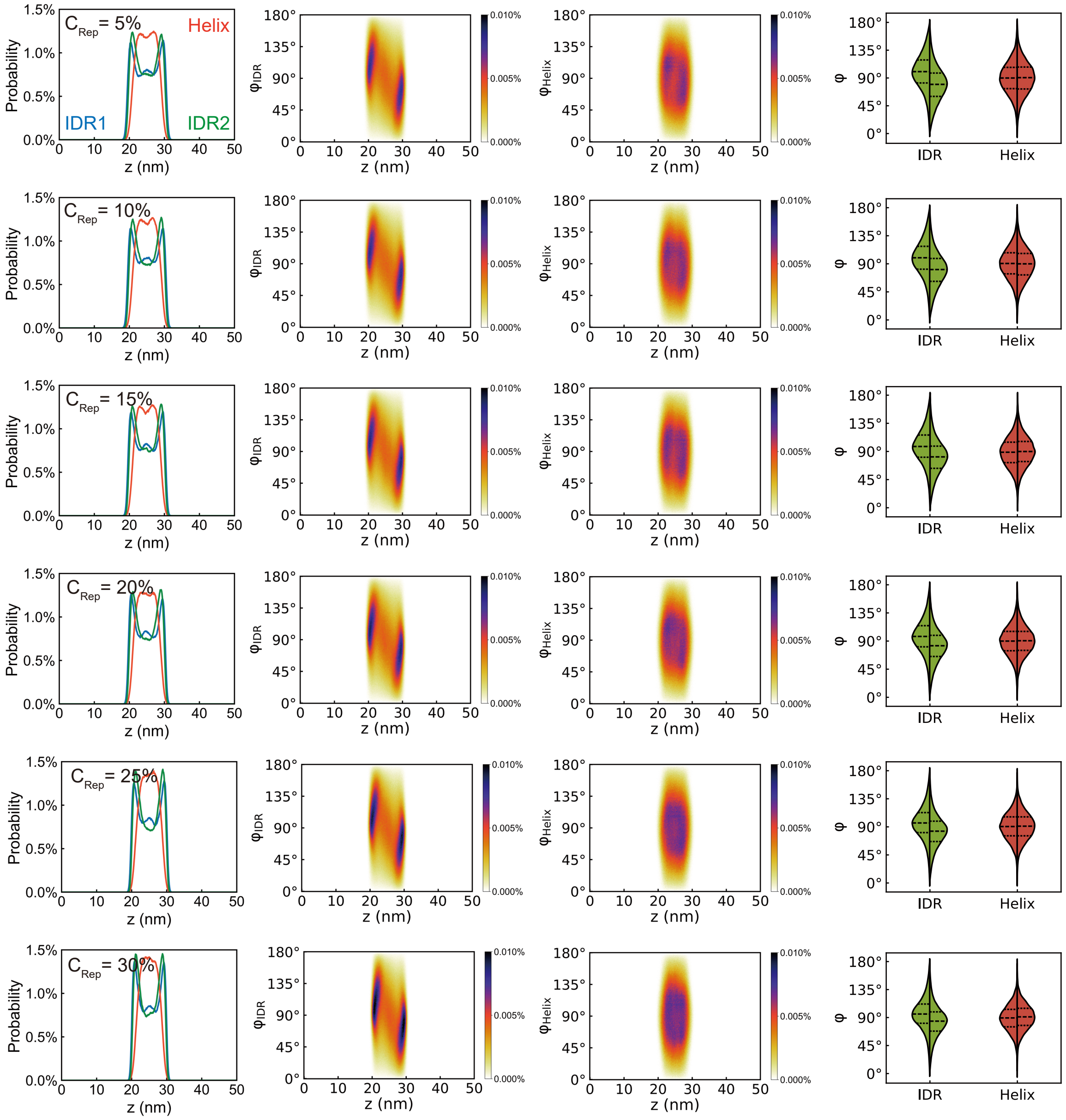

Figure S11: Region-specific spatial and orientational distributions of TDP-43 CTD segments within condensates under varying concentrations of repulsive crowders. Format and analysis are consistent with Figure 3 in the main text.

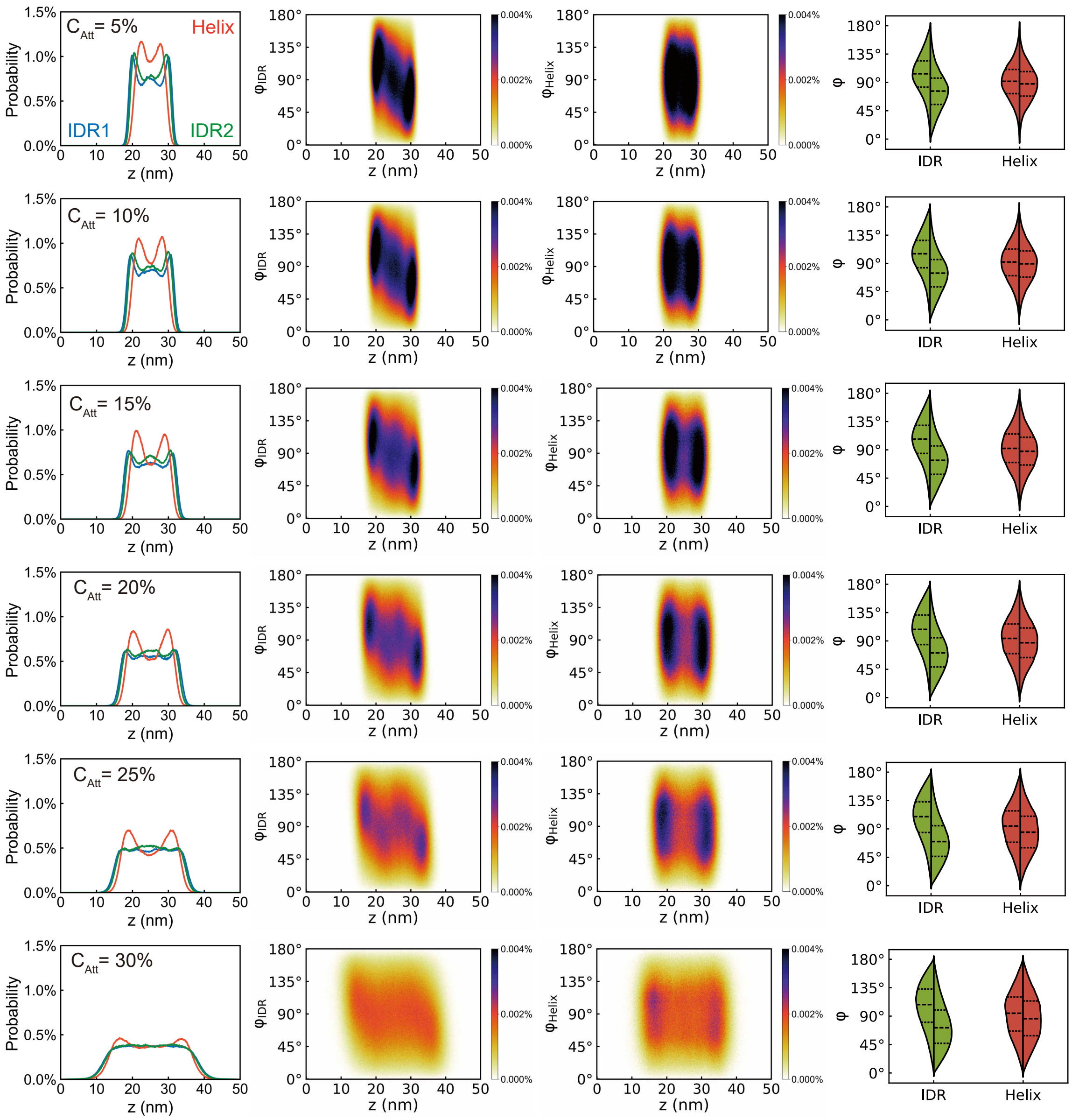

Figure S12: Region-specific spatial and orientational distributions of TDP-43 CTD segments within condensates under varying concentrations of attractive crowders. Format and analysis are consistent with Figure 3 in the main text.

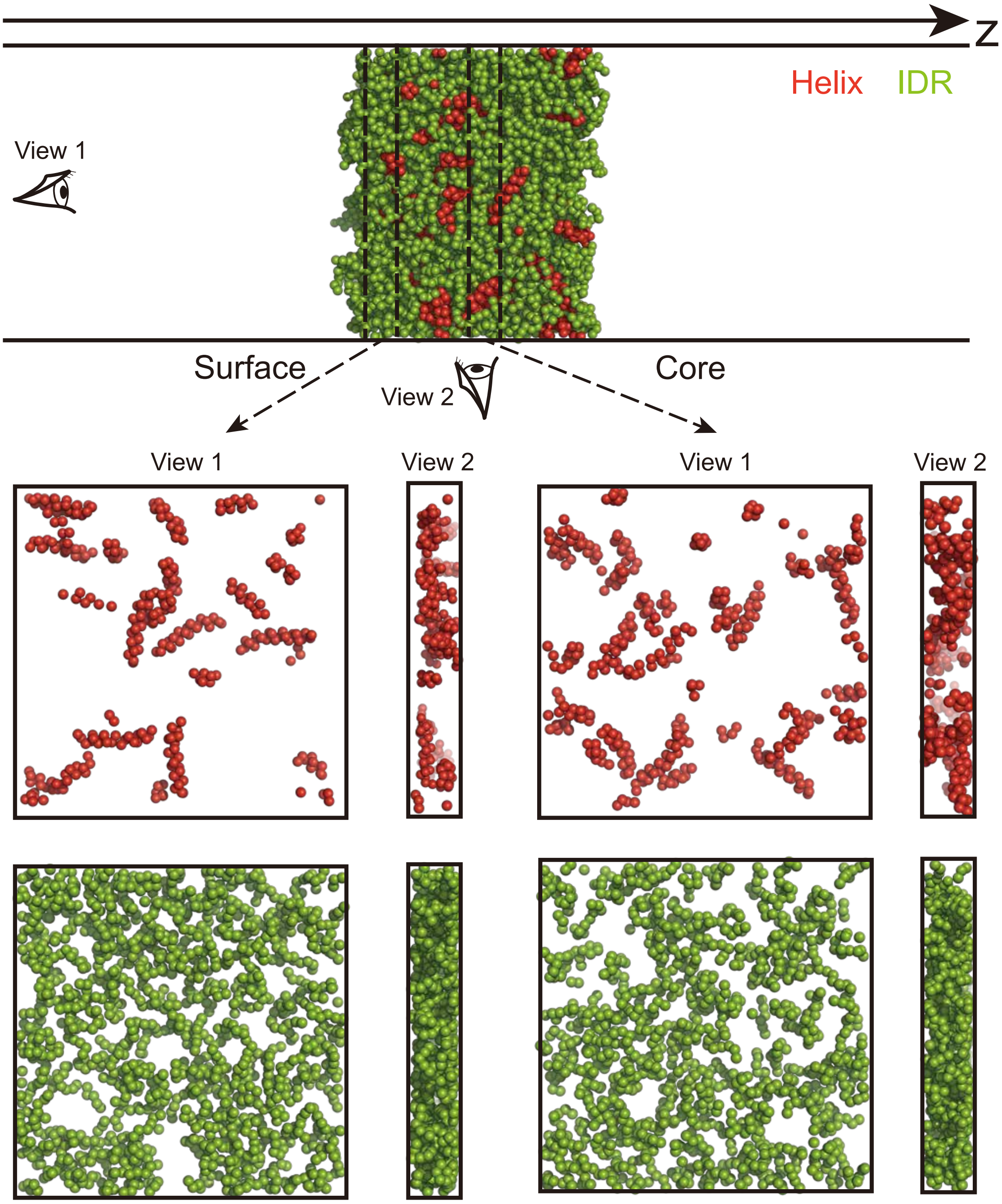

Figure S13: Representative simulation snapshots illustrating the spatial localization of TDP-43 CTD segments within condensates under crowder-free conditions. The α-helical (red) and IDR (green) segments are highlighted at both the 2-nm-thick surface and core layers, shown from two views: perpendicular to the condensate surface (view 1, //z) and parallel to the condensate surface (view 2, $\perp$z).

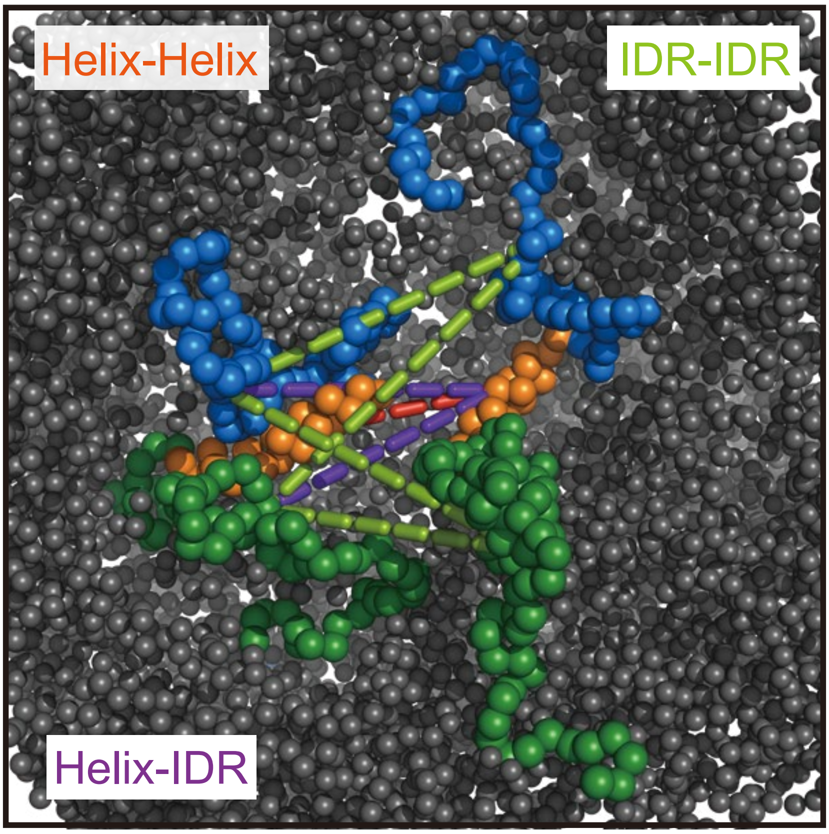

Figure S14: Schematic illustration of the three types of inter-chain region-based interactions analyzed in this study: Helix-Helix (orange), IDR-IDR (green), and Helix-IDR (purple). These interaction modes correspond to different combinations of the structured α-helical region and the flanking IDRs of TDP-43 CTD.

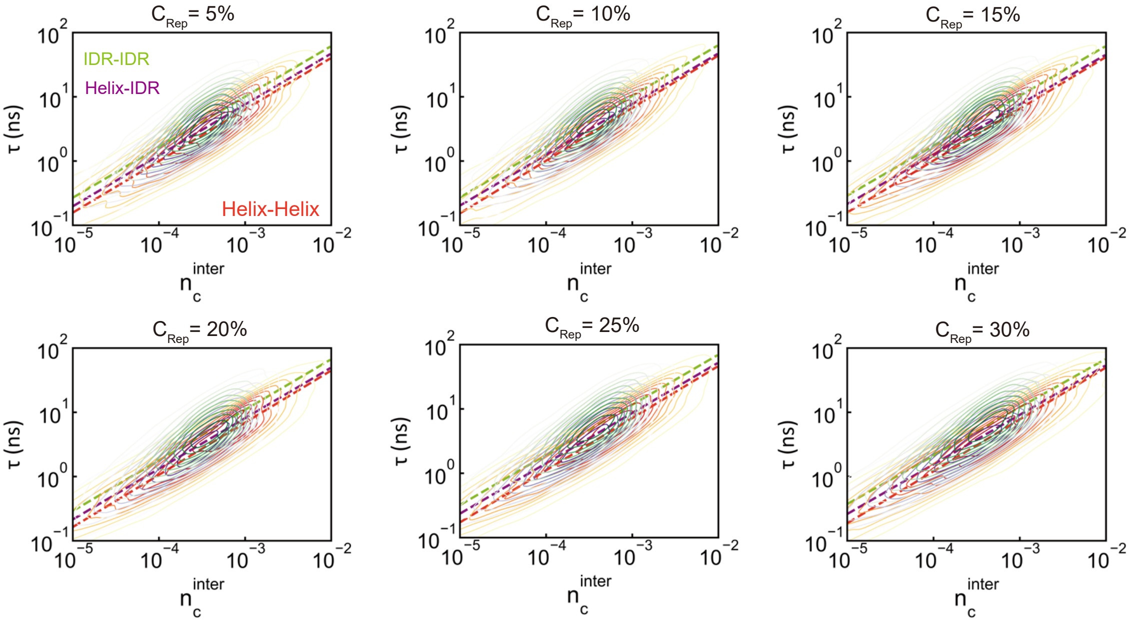

Figure S15: Two-dimensional contour plot showing the relationship between inter-chain region-based contact number (n_c^{inter}) and contact relaxation time (τ) in the presence of repulsive crowders under varying concentrations. Dashed lines indicate linear fits for each interaction type. This analysis is equivalent to that shown in Figure 4B of the main text.

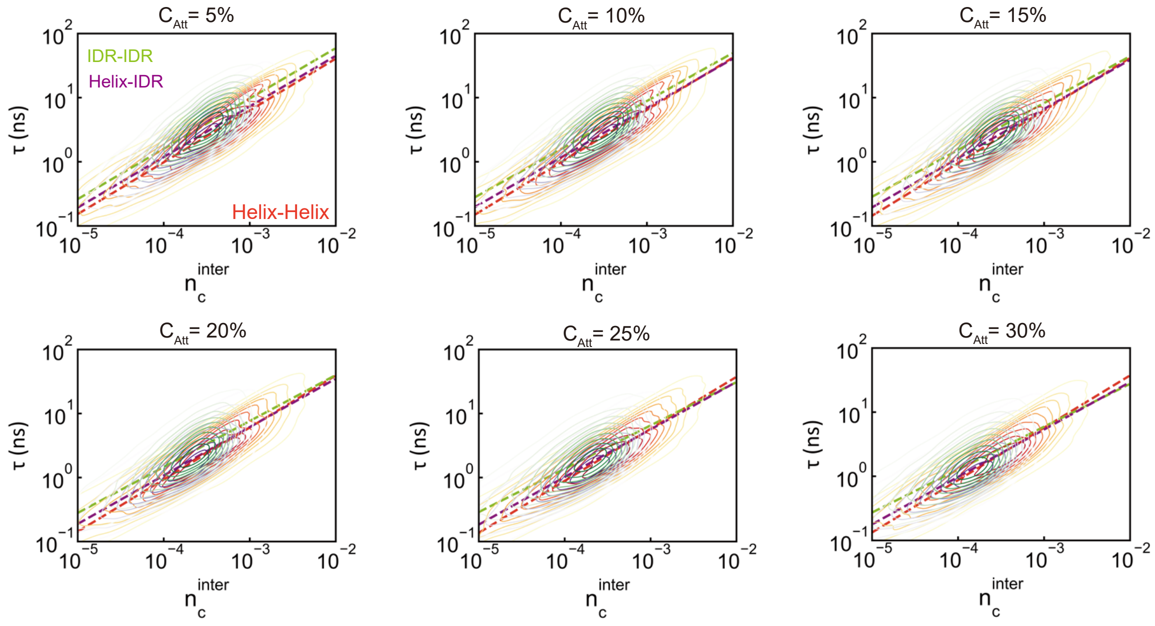

Figure S16: Two-dimensional contour plot showing the relationship between inter-chain region-based contact number (n_c^{inter}) and contact relaxation time (τ) in the presence of attractive crowders under varying concentrations. Dashed lines indicate linear fits for each interaction type. This analysis is equivalent to that shown in Figure 4B of the main text.

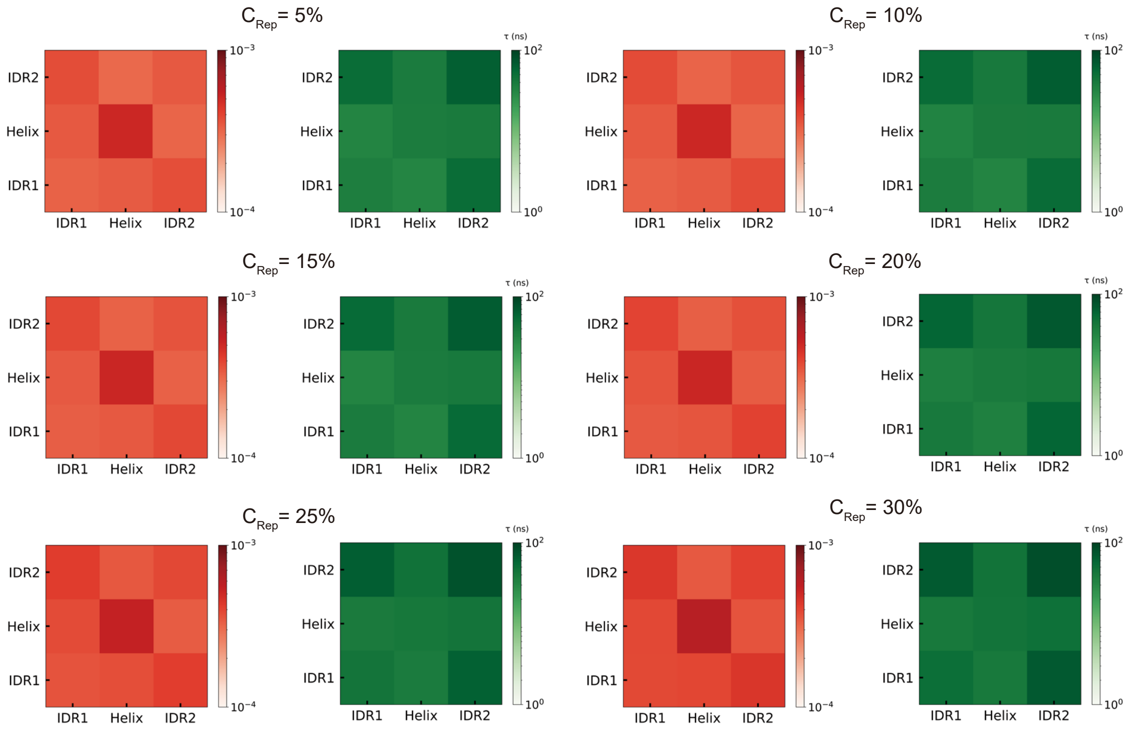

Figure S17: Heat maps showing the inter-chain region-based contact number (n_c^{inter}) and contact relaxation time (τ) under varying concentrations of repulsive crowders.

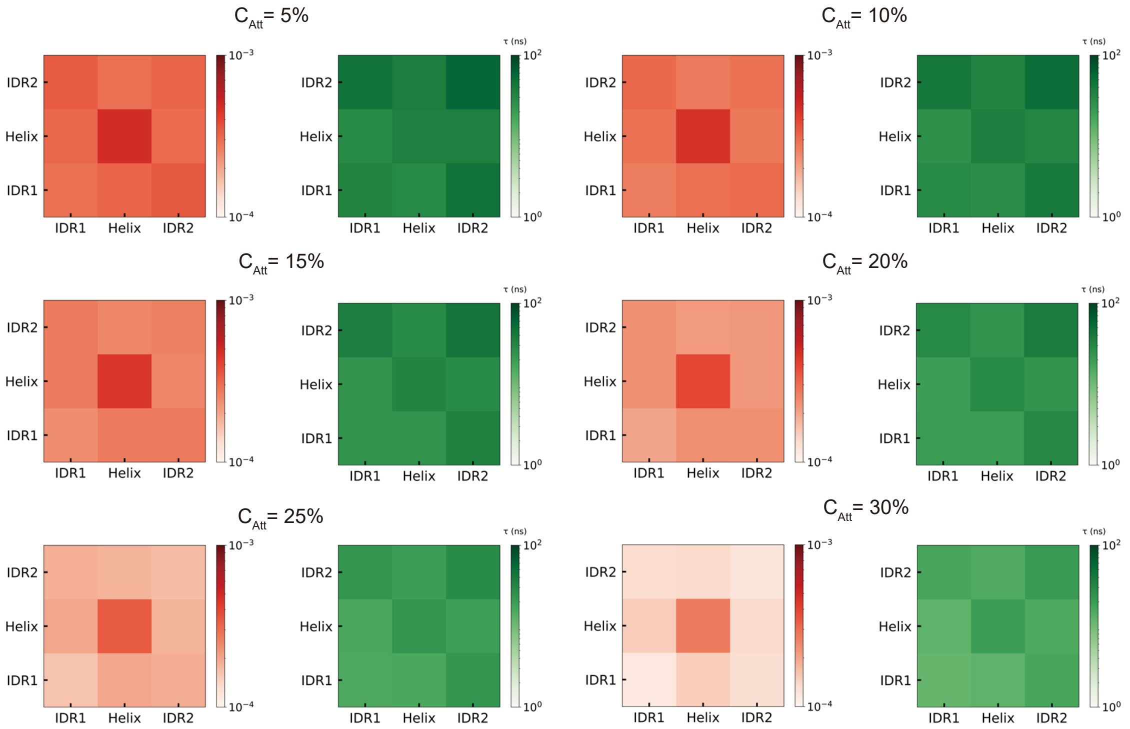

Figure S18: Heat maps showing the inter-chain region-based contact number (n_c^{inter}) and contact relaxation time (τ) under varying concentrations of attractive crowders.

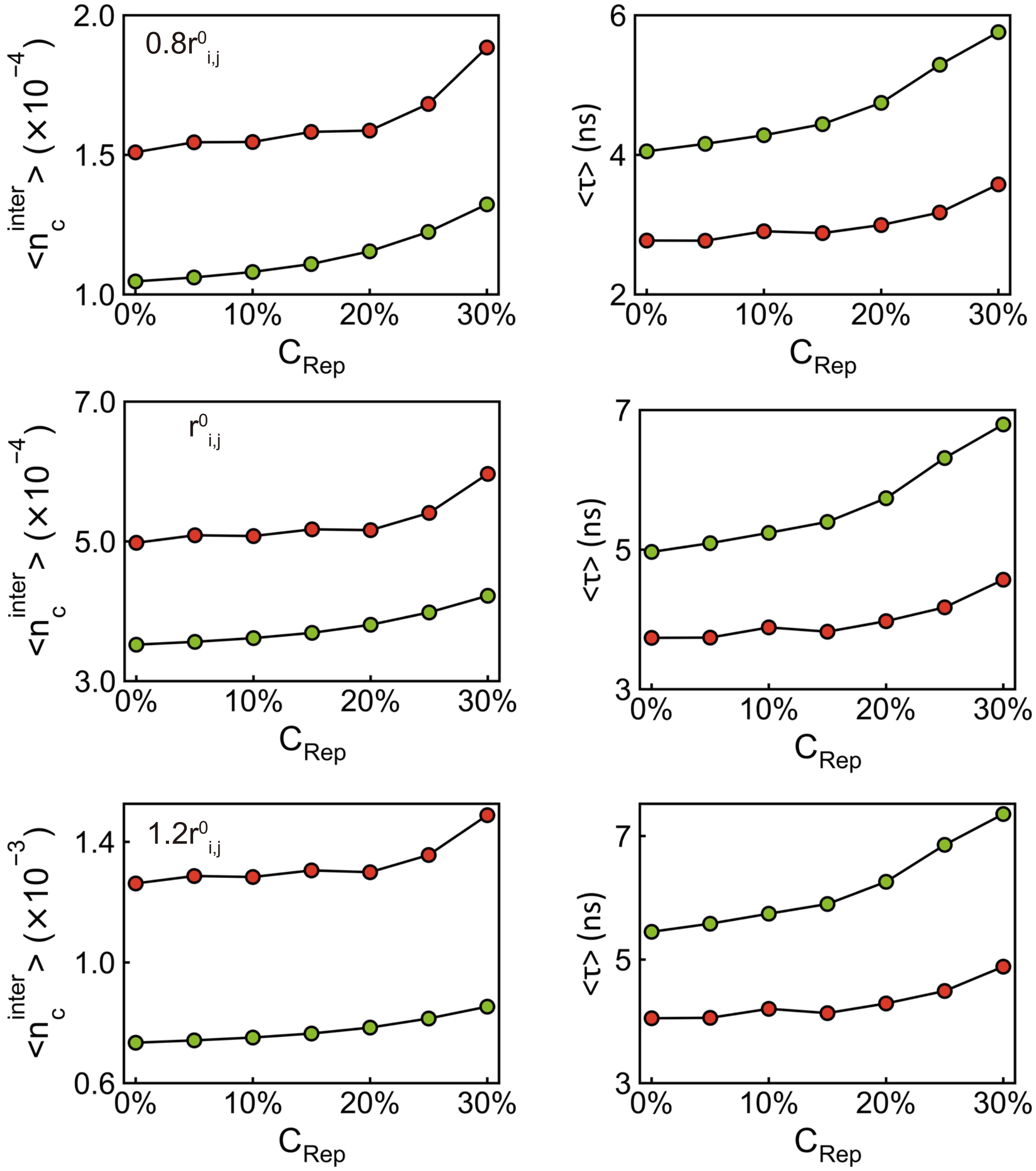

Figure S19: Inter-chain region-based contact number (n_c^{inter}) and corresponding contact relaxation time (τ) for TDP-43 CTD within condensates under varying concentrations of repulsive crowders. Values of n_c^{inter} were calculated using three different cut-off distances: $0.8r_{i,j}^0$, $r_{i,j}^0$ (default), and $1.2r_{i,j}^0$.

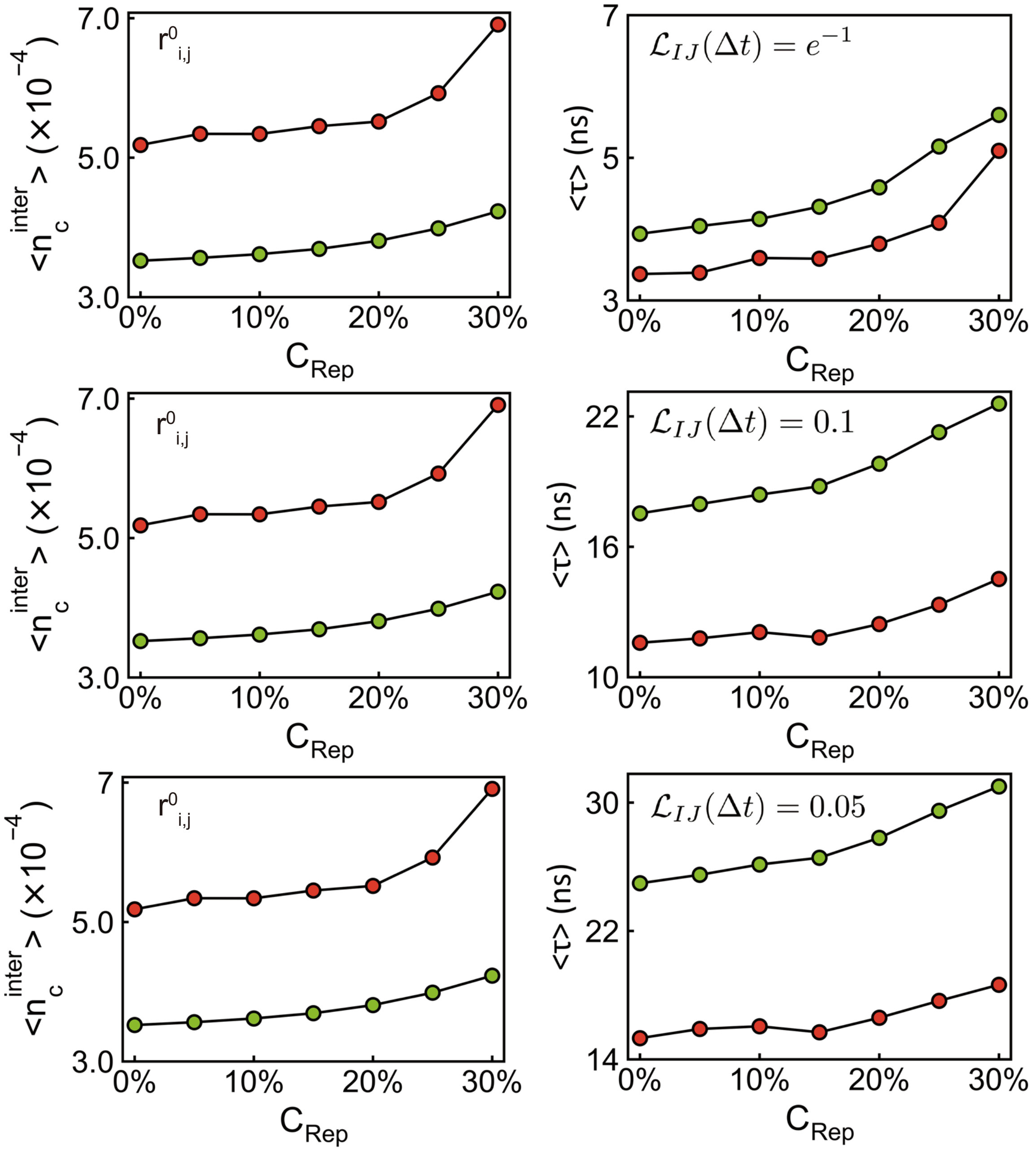

Figure S20: Inter-chain region-based contact number (n_c^{inter}) and corresponding contact relaxation time (τ) for TDP-43 CTD within condensates under varying concentrations of repulsive crowders. The relaxation time τ was obtained from the decay of the inter-region contact autocorrelation function $\mathscr{L}_{IJ}(\Delta t)$, defined as the first passage time at which $\mathscr{L}_{IJ}(\Delta t)$ falls below the threshold values e^{-1}, 0.1, or 0.05.

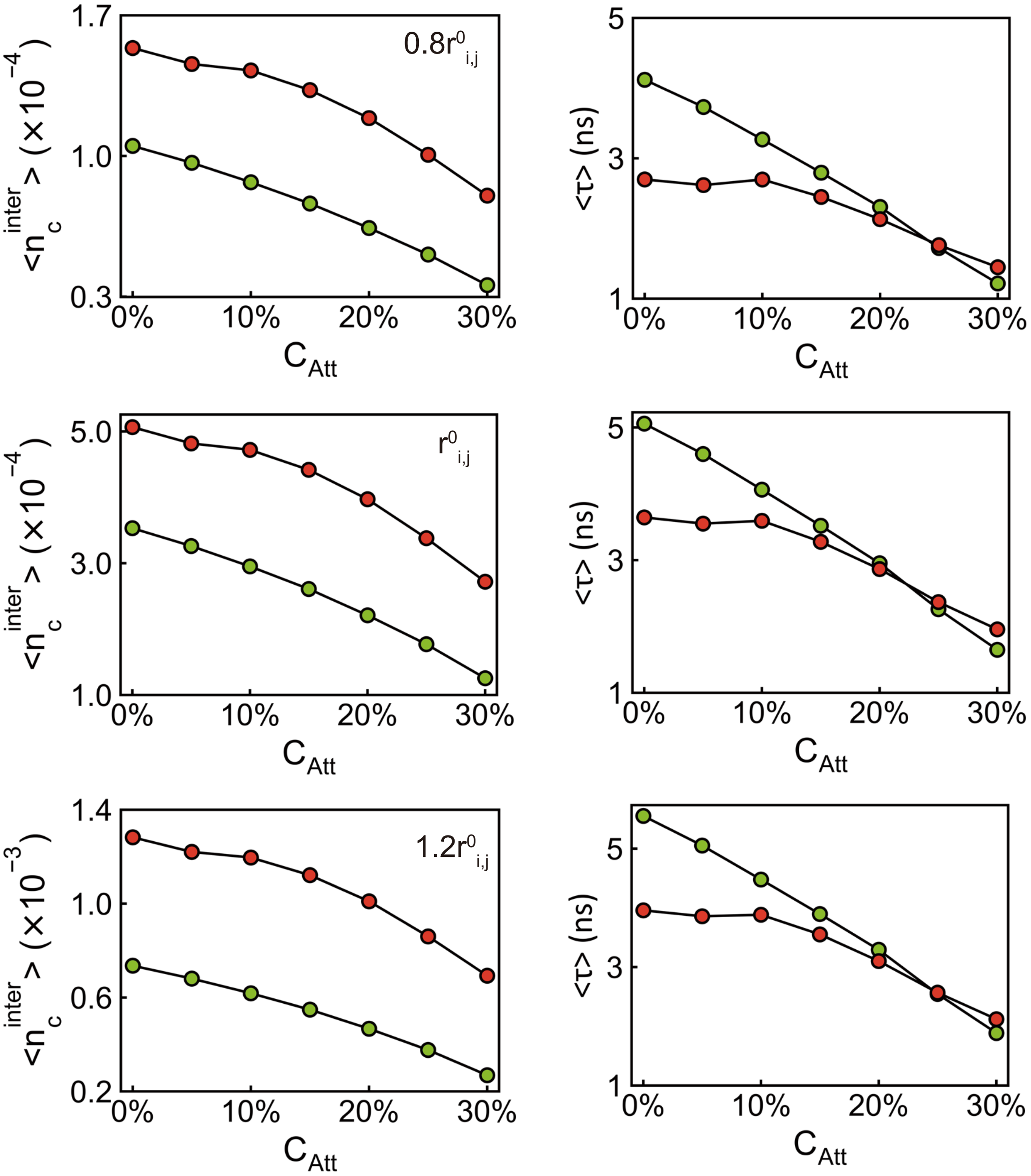

Figure S21: Inter-chain region-based contact number (n_c^{inter}) and corresponding contact relaxation time (τ) for TDP-43 CTD within condensates under varying concentrations of attractive crowders. Values of n_c^{inter} were calculated using three different cut-off distances: $0.8r_{i,j}^0$, $r_{i,j}^0$ (default), and $1.2r_{i,j}^0$.

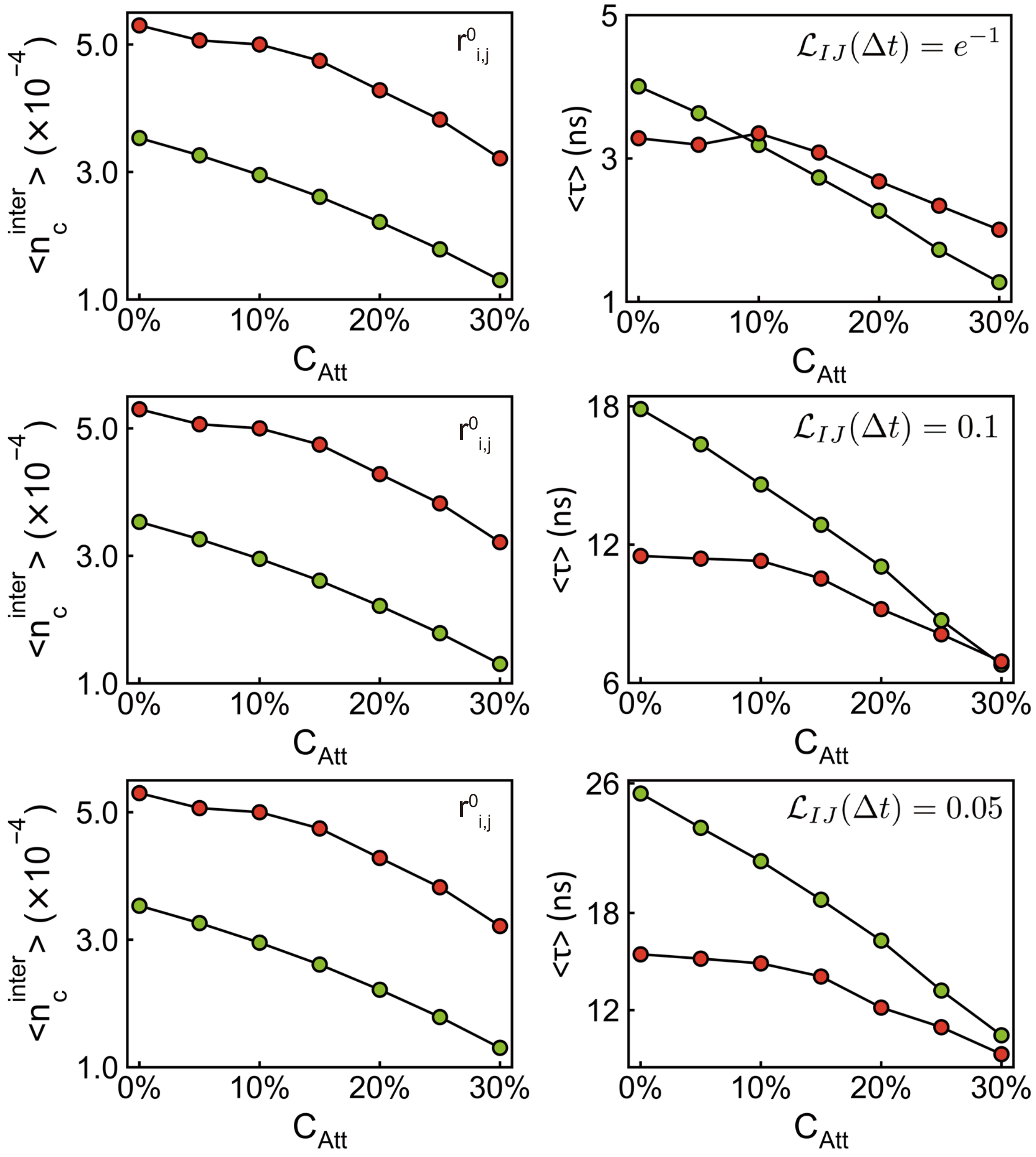

Figure S22: Inter-chain region-based contact number (n_c^{inter}) and corresponding contact relaxation time (τ) for TDP-43 CTD within condensates under varying concentrations of attractive crowders. The relaxation time τ was obtained from the decay of the inter-region contact autocorrelation function $\mathscr{L}_{IJ}(\Delta t)$, defined as the first passage time at which $\mathscr{L}_{IJ}(\Delta t)$ falls below the threshold values e^{-1}, 0.1, or 0.05.

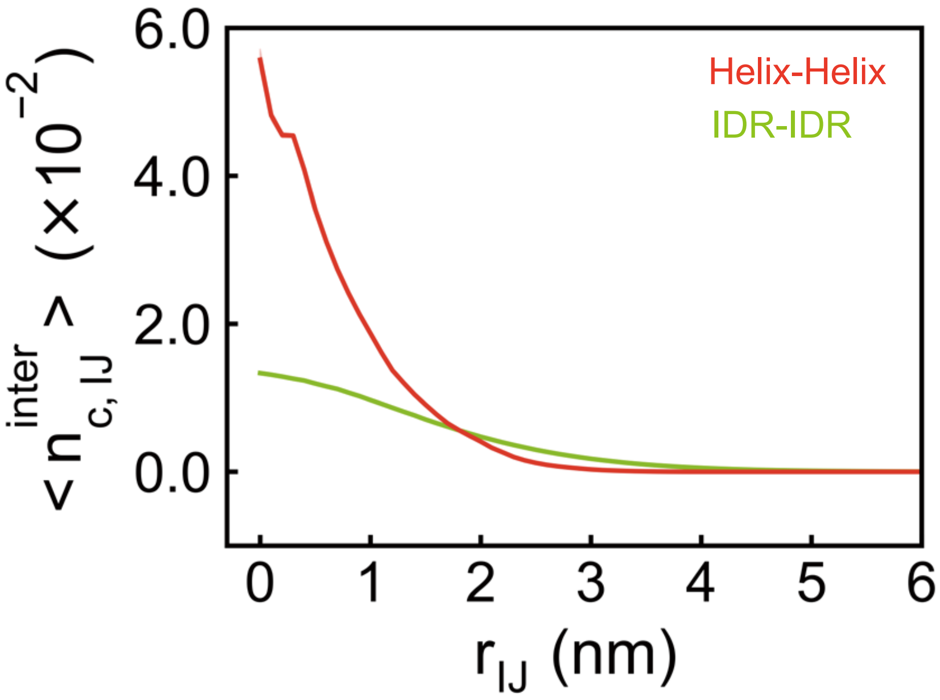

Figure S23: Average inter-chain region-based contact number ($n_{c,IJ}^{\mathrm{inter}}$) as a function of the center-of-mass distance (r_{IJ}) between two regions in the condensed phase under crowder-free conditions.

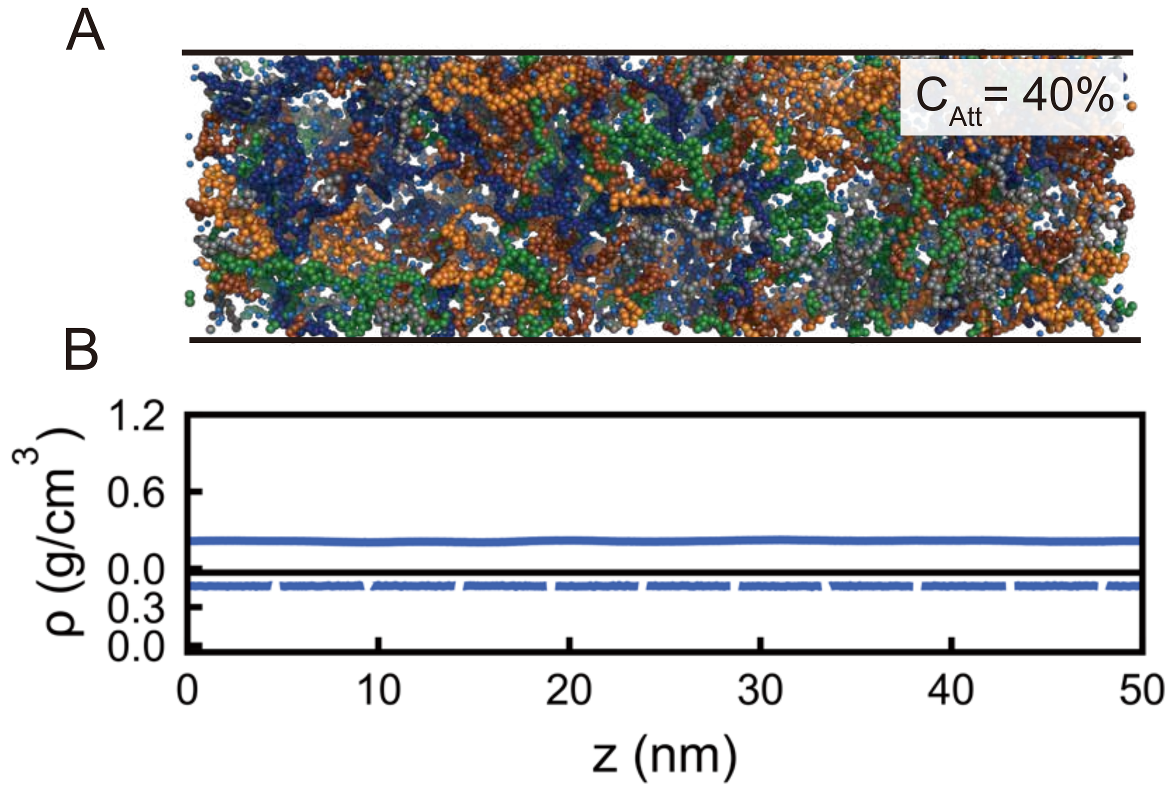

Figure S24: TDP-43 CTD phase behavior under a high concentration of attractive crowders (40% volume fraction, C_{Att}=40%, represented by blue beads). (A) Representative simulation snapshots illustrating the system morphology, where crowders form a percolated network and TDP-43 CTD chains accumulate around them. (B) Density profiles along the z-axis for TDP-43 CTD (solid line, upper panel) and crowders (dashed line, lower panel), showing that both species are uniformly distributed without the formation of a distinct protein-dense condensate.
